# Cancer immunotherapy: are we there yet?

**DOI:** 10.1186/2162-3619-2-33

**Published:** 2013-12-10

**Authors:** Zihai Li, Lieping Chen, Mark P Rubinstein

**Affiliations:** 1 Hollings Cancer Center, Medical University of South Carolina, Charleston, SC 29425, USA; 2 Yale Cancer Center, Yale University School of Medicine, New Haven, CT 06514, USA

## Abstract

The immune system is the built-in host defense mechanism against infectious agents as well as cancer. Protective immunity against cancer was convincingly demonstrated in the 1940s with syngeneic animal models (JNCI 18:769-778, 1976; Cancer Immun 1:6, 2001). Since then, the last century’s dream has been to effectively prevent and cure cancers by immunological means. This dream has slowly but surely become a reality (Nature 480:480-489, 2011). The successful examples of immunoprophylaxis and therapy against cancers include: (i) targeted therapy using monoclonal antibodies (Nat Rev Cancer 12:278-287, 2012); (ii) allogeneic hematopoietic stem cell transplantion to elicit graft-versus-cancer effect against a variety of hematopoietic malignancies (Blood 112:4371-4383, 2008); (iii) vaccination for preventing cancers with clear viral etiology such as hepatocellular carcinoma and cervical cancer (Cancer J Clin 57:7-28, 2007; NEJM 336:1855-1859, 1997); (iv) T cell checkpoint blockade against inhibitory pathways including targeting CTLA-4 and PD-1 inhibitory molecules for the treatment of melanoma and other solid tumors (NEJM 363:711-723, 2010; NEJM 366:2443-2454, 2012; NEJM 369:122-133, 2013; NEJM 366:2455-2465, 2012); (v) antigen-pulsed autologous dendritic cell vaccination against prostate cancer (NEJM 363:411-422, 2010); and (vi) the transfer of T cells including those genetically engineered with chimeric antigen receptors allowing targeting of B cell neoplasms (NEJM 365:725-733, 2011; NEJM 368:1509-1518, 2013; Blood 118:4817-4828, 2013; Sci Transl Med 5:177ra138, 2013).

This article provides an overview on the exciting and expanding immunological arsenals against cancer, and discusses critical remaining unanswered questions of cancer immunology. The inherent specificity and memory of the adaptive immune response towards cancer will undoubtedly propel cancer immunotherapy to the forefront of cancer treatment in the immediate near future. Study of the fundamental mechanisms of the immune evasion of cancer shall also advance the field of immunology towards the development of effective immunotherapeutics against a wide spectrum of human diseases.

## Introduction

Cancer immunotherapy has come a long way [1-16]. In the late 1800 s, William Coley was one of a growing number of investigators who noticed a correlation between regression of cancer and infection [17-20]. Coley expanded on this observation and became the first person to treat substantial numbers of cancer patients with a mixture of killed bacteria (known as Coley’s toxin). Although not meeting the standards of today’s trials, Coley achieved tumor regression in a relatively high proportion of sarcoma patients. Despite much enthusiasm, the advent of immune-suppressing radiation therapy and chemotherapy which could directly impact cancer progression diverted much attention away from immune-based therapies [17,18]. Furthermore, as the immune system was not well understood, there was much skepticism that tumor cells could be different from self and capable of eliciting immune-mediated eradiation. However, with growing understanding of how the immune system functioned, in 1957, Frank Macfarlane Burnet proposed a revolutionary concept that cancer cells may have antigenic differences allowing immune-mediated eradication [21]. This seed of great expectation raised hope that one day cancers might be routinely and effectively treated by immunological means. While there has been much optimism over the past 50 years, it is only during the last decade that this optimism has been met with true meaningful progress [22,23]. There is now no question that cancer immunology has entered into a period of renaissance [24,25], thanks largely to the affirmative and emphatic answer to several fundamental questions: (i) does cancer immunity exist? [2] (ii) can cancer-specific immunity lead to eradication of large established cancer? [16,26] (iii) does host immune defense exert pressure to cancer during oncogenesis? [27,28] (iv) are there tumor-specific and/or tumor-associated antigens? [29-31] (v) can immune tolerance to cancer be broken to result in therapeutic benefit? [8,10,32] Therefore, it is not a question of “if” but for many cancers “when” immunotherapy will be the main treatment modality.

### Established practice of immunotherapy of cancer

Cancer immunotherapy has already entered the mainstream of oncology [23]. Existing strategies focus on enhancing immune destruction of cancer cells by a variety of means (Table 1). One of the most successful and longstanding forms of cell-based immunotherapy is allogeneic stem cell transplant for the treatment of hematological malignancies. Although stem cell transplantation was initially thought to enhance cancer cure by allowing myeloablative therapy in the forms of high dose chemotherapy and total body irradiation [33], it has become clear that allogeneic immune response against tumor cells is a key mechanism of action [5]. The antibody-based strategy against cancer continues to make impact in cancer care, as antibodies can eliminate cancer cells via immunological means (through antibody or complement-dependent cytotoxicty) as well as via other biological means (e.g., blocking key oncogenic signals) [22,34,35]. In addition, immunomodulating cytokines remain important in the treatment of selected tumor types, such as the use of type I interferon as an adjuvant therapy for high-risk melanoma [36]. One significant milestone in the field of cancer immunology was the 2010 FDA-approval of sipuleucel-T (Sip-T), an autologous dendritic cell preparation, loaded with recombinant fusion protein between GM-CSF and prostate-specific acid phosphatase, for the treatment of metastatic prostate cancer [12]. Sip-T represents the first of its kind of therapeutic vaccine against cancer using dendritic cell-based platform [37]. In 2011, the FDA approved ipilimumab injection for the treatment of unresectable or metastatic melanoma. Ipilimumab represents a new class of cancer immunotherapeutics based on blockage of negative T cell check-point signal [3,38]. Tumor-specific T cells can be activated by professional antigen presenting cells through engagement of T cell receptor and co-stimulatory molecules such as CD28. However, to maintain immune homeostasis, activated T cells have to be temporally turned off via engaging inhibitory receptors on T cells such as CTLA-4 [39,40]. Ipilimumab is a fully human monoclonal antibody that binds and blocks CTLA-4 to sustain T cell activity, and it has been shown to improve overall survival of patients with advanced melanoma [8]. Importantly, maximizing T cell co-stimulation was demonstrated to be an effective anti-tumor strategy as early as 1992 when stable expression of the B7 molecule in tumor cells was shown to result in T-cell specific tumor eradication [41].

**Table 1:** Examples of FDA-approved cancer immunotherapeutic agents

**Modality**	**Principle**
Hematopoietic stem cell transplantion (e.g., leukemia and myeloma)	1. Reset the immune system
	2. Allo-antigen response (graft versus tumor effect)
Antibody (e.g., retuximab, trastuzumab)	1. Eliminate cancer cells
	2. Block key signaling pathways
Cytokines (e.g., type I interferon, interleukin-2)	Boost both innate and adaptive immunity
Dendritic cells (e.g., Sip-T for prostate cancer)	Enhance tumor-specific T cell priming
T cell checkpoint blockade (e.g., Ipilumimab for melanoma)	Block/reverse immune tolerance
Microbes (e.g., BCG for the transitional bladder cancer)	Enhance innate and adaptive immunity

### What is hot in cancer immunotherapy in 2013?

**Figure 1:**
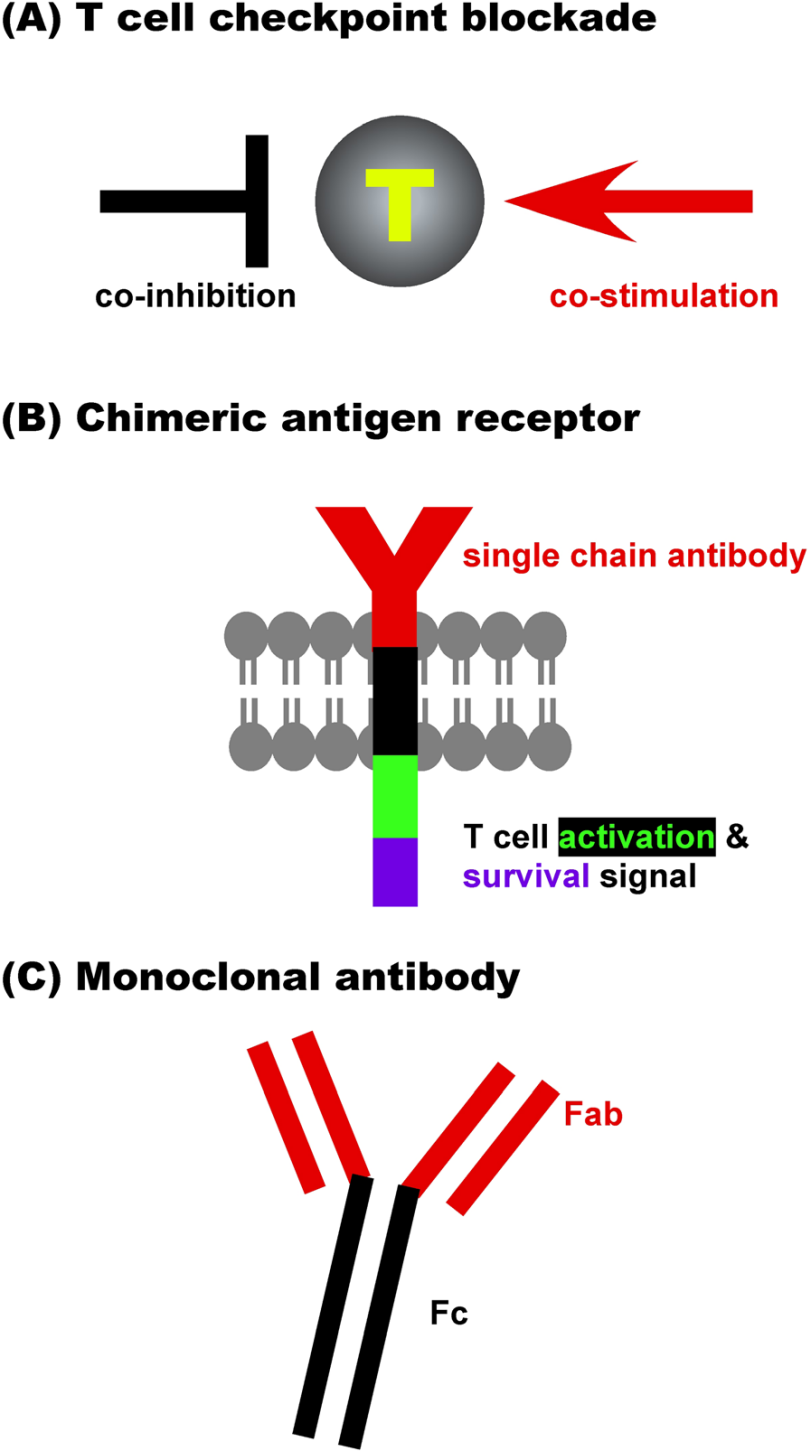
**Emerging effective immunotherapies of cancer. A**. T cell checkpoint blocker. **B**. Adoptive therapy with CAR-enforced T cells. **C**. Monoclonal antibody therapy.

Three kinds of cancer immunotherapeutics have emerged as key breakthroughs in clinical cancer care in 2013. These advances include T cell checkpoint blockers (Figure 1A), adoptive therapy with T cells genetically modified with chimeric antigen receptor (CAR) (Figure 1B), and targeted monoclonal antibody therapy (Figure 1C).

Recent findings demonstrate that a variety of functionally non-overlapping co-inhibitory receptors can be expressed by T cells to turn off their effector function [3,42]. These inhibitory receptors include CTLA-4, PD-1, TIM-3, BTLA, PD-1H (VISTA) and LAG-3. While in theory, blocking any of these inhibitory receptors could lead to increased activation of tumor-reactive T cells, systemic activation of T cells does not necessarily lead to more effective anti-tumor activity. Blockade of CTLA-4 with antibody led to tumor regression in 10-15% patients with advanced melanoma whereas severe autoimmune toxicity was evident in >30% of patients. Therefore, a strategy to selectively manipulate tumor microenvironment rather than systemic promotion of T cell immunity is desirable. It is particularly promising as many tumor cells express ligands for the co-inhibitory receptors, such as PD-L1 (also known as B7-H1), the ligand for PD-1 whereas there is minimal expression of this molecule in normal tissues [43]. Indeed, 9 of 25 patients (36%) with PD-L1-positive advanced tumors had an objective response to anti-PD-1 antibody therapy [9]. Surprisingly, when nivolumab (anti-PD-1 antibody) and ipilimumab were given to patients with advanced melanoma concurrently every 3 weeks for 4 doses, followed by nivolumab alone every 3 weeks for 4 doses, 53% of patients had an objective response, all with tumor reduction of 80% or more [10]. This study demonstrated the potential to combine multiple T cell checkpoint blockade to maximize anti-cancer immunity, with acceptable toxicity. Because this type of immunotherapy depends on a healthy immune system, conventional cancer treatments including chemotherapy and radiation therapy often impair immune system and could decrease the efficacy of this therapy. The field is waiting with anticipation of data of frontline therapy with T cell checkpoint blockade of cancer patients.

Several proof-of-principle studies have demonstrated the huge potential of utilizing synthetic immunology to engineer CAR-expressing or TCR-expressing T cells for the adoptive therapy of select cancers including lymphocytic leukemia [44-46]. Building off work from Steven Rosenberg’s group [47], Carl June and his colleagues successfully engineered a CD19-reactive CAR composed of a fusion protein between extracellular single chain anti-CD19 antibody, the transmembrane domain, 4-1BB (CD137) survival signal, and the CD3ς chain signaling motif [48]. Autologous T cells transduced with the CD19-reactive CAR were shown to have potent clinical activity against CD19^+^ tumors after infusion in three of three patients with advanced chronic lymphocytic leukemia (CLL) [13]. In April 2013, it was reported that two children with relapsed and refractory pre-B-cell acute lymphocytic leukemia received infusions of T cells transduced with CD19-reactive CAR [14]. In both patients, these T cells expanded to a level that was more than 1000 times as high as the initial engraftment level, and the cells were identified in bone marrow. In addition, the CAR^+^ T cells were observed in the cerebrospinal fluid. More importantly, complete remission was observed in both patients and is ongoing in one patient at 11 months after treatment at the time of the report. The other patient had a relapse, with blast cells that no longer expressed CD19, approximately 2 months after treatment. Thus, CAR-modified T cells are capable of killing even aggressive, treatment-refractory acute leukemia cells *in vivo*. Indeed, Michel Sadelain’s group treated five relapsed acute B cell lymphocytic leukemia subjects with autologous T cells expressing a CD19-reactive CD28/CD3ζ second-generation dual-signaling CAR [16]. All patients with persistent disease upon T cell infusion demonstrated rapid tumor eradication and achieved complete molecular remissions as assessed by polymerase chain reaction, therefore, this therapy appears to be very promising in treating hematopoietic malignancies. It will be interesting to see whether the same approach could also be applied successfully for the treatment of solid tumors.

2013 continues to witness the increasing application of monoclonal antibody for cancer immunotherapy [22]. Approved monoclonal antibodies by the FDA in 2013 include Ado-trastuzumab emtansine and pertuzumab for Her2^+^ breast cancer, denosumab for giant cell tumor of the bone, bevacizumab for both first and second line therapy for metastatic colorectal cancer in combination with chemotherapy. Ever since anti-CD3 antibody was approved in 1986 for the treatment of autoimmune diseases, more than 35 antibodies have been introduced to the market with a significant portion of them indicated for cancer therapy including rituximab (anti-CD20 antibody), Herceptin (anti-Her2 antibody), and Ipilimumab (anti-CTLA-4 antibody).

### Key unanswered questions in cancer immunology

The current enthusiasm in cancer immunology raises the question of whether all cancers may be amenable by immune intervention. As a basis for addressing this question and making existing therapies more effective, there are four critical unanswered questions (Table 2). First, what is the molecular entity in cancer that triggers the initial immune response particularly during advanced disease? If we understand the antigens the immune system targets successfully in cancers known to be immune amenable, we may be able to better identify such antigens in patients with other types of cancer. Second, what determines the outcome of tumor immunity? We need to be mindful that tumors are not bacteria, virus or parasites. The quality of immune response to cancer cannot simply be viewed through the conventional immunological prisms we use to predict the immune response against infectious agents. Third, what is the mechanism of tumor evasion? In this case, the answer might have to come from the study of the tumor microenvironment rather than systemic suppression. Finally, as much as we all hope to have a universal cancer vaccine on a population basis [49], immunotherapy of established (clinically detectable) cancer may often need to be individualized. Figuring out how to combine various arsenals in the immune system in a tailored fashion to individual patients is a challenge as well as a wonderful opportunity for future research.

**Table 2:** A few key research themes in cancer immunology

**Theme**	**Research question**
**Immune recognition of cancer**	What are characteristics of antigens critical for immune recognition of cancer cells? Do these antigens exist for all cancers?
**Fate determination of tumor immunity**	Tumors are not bacteria, not viruses, and not parasites. How do differences in antigen presentation and innate immunity signals impact the ability to initiate and mediate effective anti-tumor immunity?
**Mechanism of immune evasion**	Are immune evasion and oncogenesis closely coupled? What is the molecular definition of oncoinflammation in the tumor environment and its impact on cancer immunity?
**Immunotherapy**	It is time to redefine the goals of conventional therapy to convert non-immunogenic signals to immunogenic ones. What is the best strategy to combine immunotherapy with radiation therapy, chemotherapy or targeted therapy? More innovative immunotherapeutic strategies are needed including novel targets (e.g., cancer stem cells), novel sources of antigens (subdominant antigens), novel adjuvants, novel cytokines and new ways to reset the immune system from tolerogenic status to immunogenic one.

### Goals of cancer immunotherapy and perspective: are we there yet?

Slowly but surely there has been a growing paradigm shift in the understanding of biology and immunology of cancer. This is evident from the essay by Douglas Hanahan and Robert Weinberg on the Hallmarks of Cancer (The Next Generation) in 2011 which added “avoiding immune destruction” and “tumor-promoting inflammation” as another two hallmarks of cancer to their original perspective [50]. Given that the immune system has a remarkable ability to detect, “remember” and eliminate cancer cells, it is becoming clear that immunotherapy is not simply a means of cancer treatment. Rather, establishing long-lasting, cancer-specific immunity can be a goal to allow for curative therapy. In addition, development of effective immunotherapeutic cancer strategies requires our attention to deal with both of the immunological hallmarks of cancer: “avoiding immune destruction” and “tumor-promoting inflammation”. Presently, most of the approved cancer immunotherapeutics (Table 1) focus on maximizing immune destruction of cancers. No specific modalities are available clinically to silence “tumor-promoting inflammation”, other than antibiotics and vaccines to eliminate microbes-associated cancer. We are just starting to understand how cancer cells “avoiding immune destruction”. Defining and blunting oncoinflammation will likely prove critical in the future for achieving effective anti-cancer immune responses (Table 2).

Immunotherapy of cancer is no longer a dream. Gone is the time when elimination of cancer by immunological means was anecdotal or achievable only in animal studies. Immune-based therapies have now demonstrated efficacy in a range of clinical studies and types of cancer. Adoptive transfer of tumor-reactive T cells can cure select patients with advanced metastatic disease that have exhausted all other options. Other reagents, such as selective manipulation of T cell checkpoint blockers in cancer microenvironment, offer the possibility of off-the-shelf dosing or novel combinatorial therapies. More than ever before, the field of cancer immunology is permeated with a sense of optimism [23]. The key question today is not whether immune-based therapies will transform cancer therapy, but how will these approaches transform cancer medicine in the future.

## Competing interests

The authors declare that they have no competing interests.

## Authors’ contributions

All authors wrote, reviewed and approved the manuscript.

## References

[B1] Prehn RT Main JM Immunity to methylcholanthrene-induced sarcomas J Natl Cancer Inst 1957 18 769 778 13502695

[B2] Klein G The strange road to the tumor-specific transplantation antigens (TSTAs) Cancer Immun 2001 1 6 12747767

[B3] Mellman I Coukos G Dranoff G Cancer immunotherapy comes of age Nature 2011 480 7378 480 489 10.1038/nature10673 22193102 PMC3967235

[B4] Scott AM Wolchok JD Old LJ Antibody therapy of cancer Nat Rev Cancer 2012 12 4 278 287 10.1038/nrc3236 22437872

[B5] Kolb HJ Graft-versus-leukemia effects of transplantation and donor lymphocytes Blood 2008 112 12 4371 4383 10.1182/blood-2008-03-077974 19029455

[B6] Saslow D Castle PE Cox JT Davey DD Einstein MH Ferris DG Goldie SJ Harper DM Kinney W Moscicki AB American cancer society guideline for human papillomavirus (HPV) vaccine use to prevent cervical cancer and its precursors CA Cancer J Clin 2007 57 1 7 28 10.3322/canjclin.57.1.7 17237032

[B7] Chang MH Chen CJ Lai MS Hsu HM Wu TC Kong MS Liang DC Shau WY Chen DS Universal hepatitis B vaccination in Taiwan and the incidence of hepatocellular carcinoma in children: Taiwan childhood hepatoma study group N Engl J Med 1997 336 26 1855 1859 10.1056/NEJM199706263362602 9197213

[B8] Hodi FS O’Day SJ McDermott DF Weber RW Sosman JA Haanen JB Gonzalez R Robert C Schadendorf D Hassel JC Improved survival with ipilimumab in patients with metastatic melanoma N Engl J Med 2010 363 8 711 723 10.1056/NEJMoa1003466 20525992 PMC3549297

[B9] Topalian SL Hodi FS Brahmer JR Gettinger SN Smith DC McDermott DF Powderly JD Carvajal RD Sosman JA Atkins MB Safety, activity, and immune correlates of anti-PD-1 antibody in cancer N Engl J Med 2012 366 26 2443 2454 10.1056/NEJMoa1200690 22658127 PMC3544539

[B10] Wolchok JD Kluger H Callahan MK Postow MA Rizvi NA Lesokhin AM Segal NH Ariyan CE Gordon RA Reed K Nivolumab plus ipilimumab in advanced melanoma N Engl J Med 2013 369 2 122 133 10.1056/NEJMoa1302369 23724867 PMC5698004

[B11] Brahmer JR Tykodi SS Chow LQ Hwu WJ Topalian SL Hwu P Drake CG Camacho LH Kauh J Odunsi K Safety and activity of anti-PD-L1 antibody in patients with advanced cancer N Engl J Med 2012 366 26 2455 2465 10.1056/NEJMoa1200694 22658128 PMC3563263

[B12] Kantoff PW Higano CS Shore ND Berger ER Small EJ Penson DF Redfern CH Ferrari AC Dreicer R Sims RB Sipuleucel-T immunotherapy for castration-resistant prostate cancer N Engl J Med 2010 363 5 411 422 10.1056/NEJMoa1001294 20818862

[B13] Porter DL Levine BL Kalos M Bagg A June CH Chimeric antigen receptor-modified T cells in chronic lymphoid leukemia N Engl J Med 2011 365 8 725 733 10.1056/NEJMoa1103849 21830940 PMC3387277

[B14] Grupp SA Kalos M Barrett D Aplenc R Porter DL Rheingold SR Teachey DT Chew A Hauck B Wright JF Chimeric antigen receptor-modified T cells for acute lymphoid leukemia N Engl J Med 2013 368 16 1509 1518 10.1056/NEJMoa1215134 23527958 PMC4058440

[B15] Brentjens RJ Riviere I Park JH Davila ML Wang X Stefanski J Taylor C Yeh R Bartido S Borquez-Ojeda O Safety and persistence of adoptively transferred autologous CD19-targeted T cells in patients with relapsed or chemotherapy refractory B-cell leukemias Blood 2013 118 18 4817 4828 21849486 10.1182/blood-2011-04-348540 PMC3208293

[B16] Brentjens RJ Davila ML Riviere I Park J Wang X Cowell LG Bartido S Stefanski J Taylor C Olszewska M CD19-targeted T cells rapidly induce molecular remissions in adults with chemotherapy-refractory acute lymphoblastic leukemia Sci Transl Med 2013 5 177 177ra138 10.1126/scitranslmed.3005930 PMC3742551 23515080

[B17] McCarthy EF The toxins of William B Coley and the treatment of bone and soft-tissue sarcomas Iowa Orthop J 2006 26 154 158 16789469 PMC1888599

[B18] Hoption Cann SA Van Netten JP Van Netten C Glover DW Spontaneous regression: a hidden treasure buried in time Med Hypotheses 2002 58 2 115 119 10.1054/mehy.2001.1469 11812185

[B19] Hobohm U Fever and cancer in perspective Cancer Immunol Immunother 2001 50 8 391 396 11726133 10.1007/s002620100216 PMC11032960

[B20] Goldstein MG Li Z Heat-shock proteins in infection-mediated inflammation-induced tumorigenesis J Hematol Oncol 2009 2 5 10.1186/1756-8722-2-5 19183457 PMC2644312

[B21] Burnet M Cancer; a biological approach: I: the processes of control Br Med J 1957 1 5022 779 786 10.1136/bmj.1.5022.779 13404306 PMC1973174

[B22] Sliwkowski MX Mellman I Antibody therapeutics in cancer Science 2013 341 6151 1192 1198 10.1126/science.1241145 24031011

[B23] DeVita VT Jr Rosenberg SA Two hundred years of cancer research N Engl J Med 2012 366 23 2207 2214 10.1056/NEJMra1204479 22646510 PMC6293471

[B24] Sawyers CL Abate-Shen C Anderson KC Barker A Baselga J Berger NA Foti M Jemal A Lawrence TS Li CI Cancer progress report 2013 Clin Cancer Res 2013 19 20 Suppl S4 98 24045178 10.1158/1078-0432.CCR-13-2107

[B25] Eggermont AM Kroemer G Zitvogel L Immunotherapy and the concept of a clinical cure Eur J Cancer 2013 49 14 2965 2967 10.1016/j.ejca.2013.06.019 23890942

[B26] Morgan RA Dudley ME Wunderlich JR Hughes MS Yang JC Sherry RM Royal RE Topalian SL Kammula US Restifo NP Cancer regression in patients after transfer of genetically engineered lymphocytes Science 2006 314 5796 126 129 10.1126/science.1129003 16946036 PMC2267026

[B27] Smyth MJ Godfrey DI Trapani JA A fresh look at tumor immunosurveillance and immunotherapy Nat Immunol 2001 2 4 293 299 10.1038/86297 11276199

[B28] Schreiber RD Old LJ Smyth MJ Cancer immunoediting: integrating immunity’s roles in cancer suppression and promotion Science 2011 331 6024 1565 1570 10.1126/science.1203486 21436444

[B29] Boon T Coulie PG Van den Eynde BJ van der Bruggen P Human T cell responses against melanoma Annu Rev Immunol 2006 24 175 208 10.1146/annurev.immunol.24.021605.090733 16551247

[B30] Vigneron N Stroobant V Van den Eynde BJ van der Bruggen P Database of T cell-defined human tumor antigens: the 2013 update Cancer Immun 2013 13 15 23882160 PMC3718731

[B31] Liu B Nash J Runowicz C Swede H Stevens R Li Z Ovarian cancer immunotherapy: opportunities, progresses and challenges J Hematol Oncol 2010 3 7 10.1186/1756-8722-3-7 20146807 PMC2831814

[B32] Overwijk WW Theoret MR Finkelstein SE Surman DR De Jong LA Vyth-Dreese FA Dellemijn TA Antony PA Spiess PJ Palmer DC Tumor regression and autoimmunity after reversal of a functionally tolerant state of self-reactive CD8+ T cells J Exp Med 2003 198 4 569 580 10.1084/jem.20030590 12925674 PMC2194177

[B33] Thomas ED Bone marrow transplantation: a review Semin Hematol 1999 36 4 Suppl 7 95 103 10595758

[B34] Ferris RL Jaffee EM Ferrone S Tumor antigen-targeted, monoclonal antibody-based immunotherapy: clinical response, cellular immunity, and immunoescape J Clin Oncol 2010 28 28 4390 4399 10.1200/JCO.2009.27.6360 20697078 PMC2954137

[B35] Pandey JP Li Z The forgotten tale of immunoglobulin allotypes in cancer risk and treatment Exp Hematol Oncol 2013 2 1 6 10.1186/2162-3619-2-6 23425356 PMC3598368

[B36] Agarwala SS An update on pegylated IFN-alpha2b for the adjuvant treatment of melanoma Expert Rev Anticancer Ther 2012 12 11 1449 1459 10.1586/era.12.120 23249109

[B37] Steinman RM Mellman I Immunotherapy: bewitched, bothered, and bewildered no more Science 2004 305 5681 197 200 10.1126/science.1099688 15247468

[B38] Zou W Chen L Inhibitory B7-family molecules in the tumour microenvironment Nat Rev Immunol 2008 8 6 467 477 10.1038/nri2326 18500231

[B39] Krummel MF Allison JP CD28 and CTLA-4 have opposing effects on the response of T cells to stimulation J Exp Med 1995 182 2 459 465 10.1084/jem.182.2.459 7543139 PMC2192127

[B40] Leach DR Krummel MF Allison JP Enhancement of antitumor immunity by CTLA-4 blockade Science 1996 271 5256 1734 1736 10.1126/science.271.5256.1734 8596936

[B41] Chen L Ashe S Brady WA Hellstrom I Hellstrom KE Ledbetter JA McGowan P Linsley PS Costimulation of antitumor immunity by the B7 counterreceptor for the T lymphocyte molecules CD28 and CTLA-4 Cell 1992 71 7 1093 1102 10.1016/S0092-8674(05)80059-5 1335364

[B42] Pardoll DM The blockade of immune checkpoints in cancer immunotherapy Nat Rev Cancer 2012 12 4 252 264 10.1038/nrc3239 22437870 PMC4856023

[B43] Dong H Strome SE Salomao DR Tamura H Hirano F Flies DB Roche PC Lu J Zhu G Tamada K Tumor-associated B7-H1 promotes T-cell apoptosis: a potential mechanism of immune evasion Nat Med 2002 8 8 793 800 12091876 10.1038/nm730

[B44] Restifo NP Dudley ME Rosenberg SA Adoptive immunotherapy for cancer: harnessing the T cell response Nat Rev Immunol 2012 12 4 269 281 10.1038/nri3191 22437939 PMC6292222

[B45] Riddell SR Jensen MC June CH Chimeric antigen receptor–modified T cells: clinical translation in stem cell transplantation and beyond Biol Blood Marrow Transplant 2013 19 1 Suppl S2 S5 23085599 10.1016/j.bbmt.2012.10.021 PMC3654529

[B46] Kershaw MH Westwood JA Darcy PK Gene-engineered T cells for cancer therapy Nat Rev Cancer 2013 13 8 525 541 10.1038/nrc3565 23880905

[B47] Kochenderfer JN Wilson WH Janik JE Dudley ME Stetler-Stevenson M Feldman SA Maric I Raffeld M Nathan DA Lanier BJ Eradication of B-lineage cells and regression of lymphoma in a patient treated with autologous T cells genetically engineered to recognize CD19 Blood 2010 116 20 4099 4102 10.1182/blood-2010-04-281931 20668228 PMC2993617

[B48] Kalos M June CH Adoptive T cell transfer for cancer immunotherapy in the era of synthetic biology Immunity 2013 39 1 49 60 10.1016/j.immuni.2013.07.002 23890063 PMC3809038

[B49] Li Y Zeng H Xu RH Liu B Li Z Vaccination with human pluripotent stem cells generates a broad spectrum of immunological and clinical responses against colon cancer Stem Cells 2009 27 12 3103 3111 19816950 10.1002/stem.234

[B50] Hanahan D Weinberg RA Hallmarks of cancer: the next generation Cell 2011 144 5 646 674 10.1016/j.cell.2011.02.013 21376230

